# Structural proteomics, electron cryo-microscopy and structural modeling approaches in bacteria–human protein interactions

**DOI:** 10.1007/s00430-020-00663-5

**Published:** 2020-02-19

**Authors:** Sounak Chowdhury, Lotta Happonen, Hamed Khakzad, Lars Malmström, Johan Malmström

**Affiliations:** 1 Division of Infection Medicine, Department of Clinical Sciences, Faculty of Medicine, Lund University, 22184 Lund, Sweden; 2 Institute for Computational Science, University of Zurich, 8057 Zurich, Switzerland; 3 Swiss Institute of Bioinformatics (SIB), 1015 Lausanne, Switzerland; 4 S3IT, University of Zurich, 8057 Zurich, Switzerland

**Keywords:** Host–pathogen interaction, Proteomics, Affinity-purification mass spectrometry, Cross-linking mass spectrometry, Electron cryo-microscopy, Molecular modeling

## Abstract

A central challenge in infection medicine is to determine the structure and function of host–pathogen protein–protein interactions to understand how these interactions facilitate bacterial adhesion, dissemination and survival. In this review, we focus on proteomics, electron cryo-microscopy and structural modeling to showcase instances where affinity-purification (AP) and cross-linking (XL) mass spectrometry (MS) has advanced our understanding of host–pathogen interactions. We highlight cases where XL-MS in combination with structural modeling has provided insight into the quaternary structure of interspecies protein complexes. We further exemplify how electron cryo-tomography has been used to visualize bacterial–human interactions during attachment and infection. Lastly, we discuss how AP-MS, XL-MS and electron cryo-microscopy and -tomography together with structural modeling approaches can be used in future studies to broaden our knowledge regarding the function, dynamics and evolution of such interactions. This knowledge will be of relevance for future drug and vaccine development programs.

## Introduction

Infectious diseases are a serious health problem aggravated by the current spread of pathogens and their vectors to new niches. Concomitantly, the emergence of novel, zoonotic pathogens is rapidly increasing as well as the bacterial resistance to antibiotics [1, 2]. In fact, several of the major health organizations including the World Health Organization (WHO) have identified infectious diseases and the increasing resistance to antibiotics as a foremost global concern [3]. The increasing bacterial resistance to antibiotics threatens to make some infections untreatable and poses a major threat to modern health care as several medical procedures are dependent on effective antibiotics. Actions are needed to promote the understanding of the molecular mechanisms by which pathogens cause disease and how they modulate their host’s cellular machinery to escape immune surveillance. Equally important is the development of new treatment alternatives to antibiotics [4].

During an infection, a bacterial pathogen circumvents host’s immune defenses via highly evolved effector proteins or virulence factors that can hijack and re-wire molecular host systems. At the same time, host proteins such as immunoglobulins, proteins of the complement system and antimicrobial proteins together with cells from the host’s adaptive and innate immune system, bind to bacterial surfaces and effector proteins to neutralize bacteria and prevent infection. This dynamic interplay between host and pathogen partly depends on the formation of host–pathogen protein–protein interactions (HP-PPIs) [5, 6]. Typically, HP-PPIs are dynamic and are under strong evolutionary pressure [7] to a point where the diversity can inundate the host immune system [8, 9]. Structural characterization of HP-PPIs has the potential to advance our understanding of the molecular mechanisms of infection as well as to provide new targets for drug and vaccine development [10, 11]. Integrative structural biology combining information from several complementary approaches within structural biology and biological mass spectrometry has the potential to improve the characterization of HP-PPIs (see “INFO BOX 1”) [12].

**Table:** 

**Info box 1: Integrative structural biology**
Integrative structural biology refers to the determination of structural model of a protein or protein complex using a variety of different structural methods, typically X-ray crystallography, nuclear magnetic resonance spectrometry (NMR), single-particle electron cryo-microscopy (cryoEM) and small-angle X-ray scattering [12, 13]. Traditionally, this has meant the fitting of high-resolution crystal structures into nanometer resolution cryoEM maps to generate an atomic model for a larger protein complex. Recent technological and computational developments are pushing these boundaries, and current methods such as chromatographic co-purification of protein complexes, cross-linking and hydrogen–deuterium exchange mass spectrometry, light scattering, mutagenesis and molecular modeling are adding valuable information for data interpretation [12, 14]. The individual pieces of data gathered using the different methods provide valuable restraints for the determination of the conformation(s), position and orientation of the components. The simultaneous use of such restraints can significantly improve the accuracy, precision and completeness of a given protein complex model [12].

**Fig. 1:**
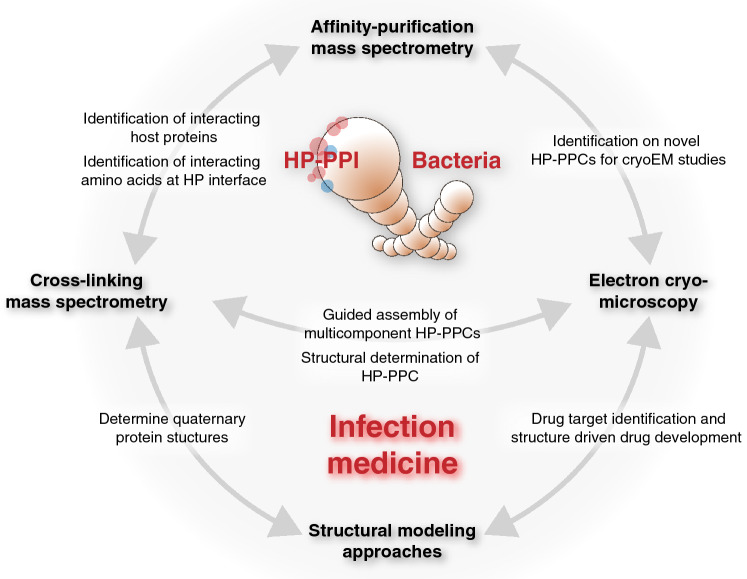
Overview of integrative proteomics, electron cryo-microscopy and structural modeling approaches in bacteria–human protein–protein interactions; *HP-PPI* host–pathogen protein–protein interaction, *HP-PPC* host–pathogen protein–protein complex, *cryoEM* electron cryo-microscopy

Successful integrative structural biology approaches for the characterization of HP-PPI networks typically requires multi-tiered information. For example, large-scale mapping of binary interspecies protein–protein interactions is important to outline the degree of interconnectivity between proteins within a network. Conversely, generation of multiple HP-PPI networks between different species will support comparative studies to promote generalized conclusions about pathogen-specific mechanisms [15–17] and common themes of interaction between different pathogen types [6, 18–21]. Such information will clarify whether particular bacterial proteins bind to more than one human protein to form larger host–pathogen protein–protein complexes (HP-PPC); or if certain human proteins are frequently targeted by several proteins from one or many bacterial pathogens [16]. Another important aspect is the knowledge of the protein–protein binding site to, for example, differentiate between direct and indirect protein interactions. In this context, highly resolved information of protein binding interfaces across species-specific HP-PPI networks has the potential to uncover underlying evolutionary conserved interaction patterns that can be further exploited for the development of new therapeutic strategies [22]. Lastly, structural information of the individual protein components as well as intact interspecies protein complexes is required to determine the structural–functional relationship of the interactions. The recent development and application of several mass spectrometry (MS)-based protein interaction analysis strategies [23] together with the ‘resolution revolution’ of cryoEM [24–26] offers new possibilities to map, characterize and functionally annotate HP-PPI networks. In this review, we highlight some recent technological developments in affinity-purification (AP) and cross-linking (XL) mass spectrometry (MS) applied together with electron cryo-tomography (cryoET) to demonstrate how these approaches have provided novel and distinct information of bacteria–human HP-PPI networks (Fig. 1). We also propose how the increasing maturity of AP-MS, XL-MS, single-particle cryoEM and cryoET is likely to advance integrative structural biology and modeling approaches.

## Affinity-purification mass spectrometry

**Fig. 2:**
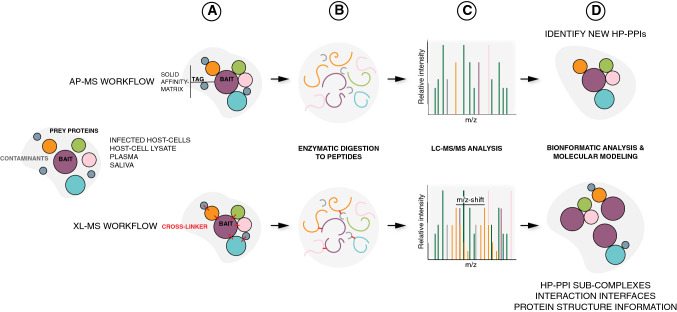
Schematic overview of the affinity-purification mass spectrometry (AP-MS) and cross-linking mass spectrometry (XL-MS) workflows. Interacting prey proteins (e.g., host proteins) to a given bait (e.g., bacterial protein) can be identified from a variety of biological mixtures, such as infected cells, host-cell lysates, plasma or saliva via AP-MS (top panel) or XL-MS (bottom panel). **a** In the AP-MS workflow, interacting prey proteins are enriched from the biological sample to an affinity-tagged bait protein attached to a solid affinity matrix; whereas in XL-MS, interacting prey proteins can be identified as associated to the bait via adding a suitable cross-linker to the sample and identifying cross-linked bait–prey peptides further down the workflow. **b** For the mass spectrometric identification of interacting proteins via either the AP-MS or the XL-MS workflow, all proteins present in either sample are digested to peptides via dedicated enzymes, prior to **c** mass spectrometric analysis of the samples via liquid chromatography tandem mass spectrometry (LC–MS/MS). In the XL-MS samples, a typical signature feature for a cross-linked peptide is an observable mass-over-charge (*m/z*) shift in the eluting peptides arising from isotopic variants of the cross-linker molecule. **d** Bioinformatic analysis of the acquired spectra allows for the identification of (novel) HP-PPIs and together with molecular modeling for the identification and structural determination of the HP-PPI sub-complexes and their interaction interfaces

AP-MS is an increasingly important technique to explore HP-PPIs based on affinity-tagged bacterial or human proteins coupled to a solid matrix to capture interacting proteins (Fig. 2 and “INFO BOX 2”). AP-MS enables the identification and quantification of multiple proteins that are enriched during the affinity purification. This technique generates information on interspecies protein–protein interactions and on occasion the dynamics of such interactions. In the broadest application, the entire proteome of a given pathogen can be analyzed—most often that of a virus—by expressing every protein as individual recombinant, affinity-tagged protein to probe proteome-wide interspecies HP-PPI [27]. Different versions of AP-MS typically rely on different data acquisition schemas and different strategies to filter out false interactions to visualize the resulting interaction network as highlighted in the examples below.

**Table:** 

**Info box 2: Affinity-purification mass spectrometry (AP-MS)**
Affinity-purification mass spectrometry (AP-MS) is based on the principle of enriching proteins (preys) or other biomolecules from a complex biological mixture using a ligand (the bait) coupled to a solid matrix via an affinity-tag (Fig. 2). The bait and the biological sample are mixed for the prey proteins to interact and bind to the bait; whereas non-interacting, unbound proteins are washed away. The bait–prey complexes are subsequently released from the solid matrix, enzymatically digested and processed for MS analysis [17, 28]. The affinity-tagged proteins are often expressed as recombinant proteins [17, 28, 29], but in case of intraspecies PPI analysis, they can equally be expressed by the recombinant cells being investigated [30–32]. Common affinity-tags used include the Strep- [17] or StrepII-tag [28, 29] and the FLAG-tag [33]. For a comprehensive review of possible tags, see Dunham et al. [34]. The captured prey proteins can be identified using various mass spectrometry acquisition methods such as data dependent acquisition (DDA) or more recently data independent acquisition (DIA) and sequential window acquisition of all theoretical mass spectra (SWATH-MS). DDA is based on the principle where the most abundant peaks in MS1 spectra within in a fixed time frame are selected to be fragmented to give rise to MS2 spectra. DIA and SWATH-MS are quite interlinked where the user defines a set range of *m/z* and allows the system to pick peaks within this set range separated by a fixed *m/z* value to be fragmented. Common data filtering algorithms for distinguishing contaminating proteins from true-positive interactors include, for instance, SAINT [35], ComPASS [36] and MiST [37].

In recent work, Happonen et al. generated a quantitative interaction map between the *Streptococcus pyogenes* bacterium and human proteins [28]. The map is composed of over 220 high-confidence HP-PPI between streptococcal virulence factors and human plasma and saliva proteins. The results demonstrated that *S. pyogenes* forms a highly interconnected HP-PPI network with human proteins, which can dynamically change in different bacterial–host microenvironments. Furthermore, the use of different *S. pyogenes* serotypes and their isogenic mutants revealed that the M1-protein, the main surface-attached virulence factor of *S. pyogenes*, interacts with many human proteins forming a large HP-PPC. These efforts provide relevant information for future vaccine development programs for *S. pyogenes* by identifying the localization of opsonizing antibodies to specific regions of the M1-protein. In another paper, Penn et al. performed an AP-MS study to identify protein interactions formed between secreted *Mycobacterium tuberculosis (Mtb)* proteins and proteins from human macrophages [29]. The study generated a global map of 187 high-confidence HP-PPI from 34 secreted *Mtb* proteins. This enabled the identification of a specific interaction between the probable conserved lipoprotein LpqN (a secreted *Mtb* virulence factor) and the ubiquitin ligase CBL. The identification of the interaction between CBL and LpqN infers a host defense mechanism limiting the growth of *Mtb* in macrophages. In a third study, Mirrashidi et al. [17] used inclusion membrane proteins (Inc) from *Chlamydia trachomatis* to generate an extensive HP-PPI network composed of 354 high-confidence lnc–human interactions. The study identified several proteins and pathways known to be modulated during infection and revealed cellular processes possibly modulated by *C. trachomatis*. Importantly, several of these HP-PPIs were found to be conserved also in viruses, such as HIV [17, 27].

These above examples highlight how AP-MS coupled with appropriate bioinformatics data analysis strategies can determine interspecies PPIs, provide evidence that several of the characterized HP-PPI networks are highly interconnected and that certain bacterial proteins can bind to several human proteins to potentially form larger inter-species protein complexes [16, 17, 28]. One of the challenges with large-scale AP-MS experiments is to filter out biological meaningful interactions from proteins that bind in an unspecific manner to the bait or the affinity matrix. It can be anticipated, however, that the generation of additional HP-PPI networks from other species will grant access to ‘‘gold-standard’’ datasets of known host–pathogen interactions that can be used to optimize a score threshold for removal of false protein interactions. In this context, novel quantitative MS-based strategies have already shown to be beneficial for discriminating between true and false interactions and has furthermore provided new opportunities to reliably quantify temporal changes of protein interaction networks [38].

## Cross-linking mass spectrometry (XL-MS)

Understanding the quaternary structure of molecular complexes at a proteome level and close to in vivo conditions holds the potential of improving our understanding of HP-PPIs. Additionally, information regarding protein interaction sites and direct and indirect protein binding within HP-PPIs provides critically important information to establish the organization and topology of the HP-PPI networks and the arising HP-PPCs [28, 39]. Cross-linking mass spectrometry (XL-MS) provides valuable information about the structural characteristics of a protein or protein complex (Fig. 2, and “INFO BOX 3”). The reactive sides of the cross-linker reagents are separated by a fixed distance. Thus, identification of cross-linked peptides provides information of the physical proximity of protein secondary structure elements, subunits and domains. This distance can then be used for molecular modelling of protein complexes [39–41]. Recent improvements in the chemical cross-linking reagents, mass spectrometers and database search algorithms [42] have improved the analysis of cross-linked peptides in complex biological samples such as cell lysates [43]. Advanced XL-MS techniques could also play an important role in integrative structural biology [14] as highlighted in the examples below.

**Table:** 

**Info box 3: Cross-linking mass spectrometry (XL-MS)**
Cross-linking mass spectrometry (XL-MS) provides evidence of proteins interacting with each other by covalently linking MS detectable amino acid pairs together [44] (Fig. 2). Commonly used cross-linkers can either be homo-bifunctional linking two identical amino acids together or hetero-bifunctional linking two different amino acids together. Different cross-linkers have different lengths of cross-linker spacer arms between the reactive groups. In the simplest case, the cross-linker is added to the biological mixture containing the proteins of interest, allowed to react with the proteins, with subsequent enzymatic digestion of the cross-linked proteins to peptides followed by MS analysis (Fig. 2). Importantly, cross-linking can equally well be applied to AP-MS samples during the experimental setup, cross-linking bait and prey proteins. As in the case of AP-MS (see “INFO BOX 2”), different MS acquisition techniques can also be used to acquire information on cross-linked peptide pairs. Current state-of-the-art data analysis pipelines and software for XL-MS data analysis (recently reviewed by Yu and Huang [46]), include the TX-MS pipeline [39], the MeroX [47] and Mass Spec Studio (https://www.msstudio.ca/) software.

In a study by Schweppe et al. [48], cross-linking *Acinetobacter baumannii* cells with human lung epithelial cells led to the first large-scale HP-PPI analysis for *A. baumannii.* Biotin-aspartate proline-PIR n-hydroxyphthalimide (BDP-NHP) was used to crosslink *A. baumannii* with human cells, followed by tryptic digestion of the cross-linked proteins and subsequent MS analysis. This cross-linking experiment using human lung epithelial cells identified the outer membrane protein A (OmpA) as a virulence factor. OmpA was found to bind to a desmosomal protein providing evidence for a novel mechanism of how *A. baumannii* enables epithelial intrusion and cell invasion. In more recent work, Hauri et al. developed a novel XL-MS workflow termed targeted chemical cross-linking (TX-MS). TX-MS relies on a combination of chemical cross-linking, high-resolution mass spectrometry and high-accuracy protein structure modeling [39]. TX-MS was used to construct a high-resolution interspecies quaternary model of the *S. pyogenes* M1–human protein complex identified in a previous AP-MS study [28], as discussed briefly above. The model explains how the repeat regions of the streptococcal M1-protein bind to several plasma proteins along its length to prevent phagocytosis, inhibit complement activation and to secure nutrients [49]. At the same time, the model also explains how *S. pyogenes* masks its conserved and vulnerable surface epitopes in the binding interfaces with human proteins [50].

The above instances demonstrate that XL-MS is a promising technique that promotes the analysis of HP-PPI networks and elucidates the arising protein complex structures. Addition of cross-linkers to proteins stabilizes their interactions in native conditions, thus providing valuable information on their dynamics and flexible regions. Moreover, covalent bond formation between interacting proteins leads to capture of weak or transient interactions thereby reducing non-specific background [43, 45]. The work using TX-MS revealed that the full structure of the M1-protein is engaged in protein interactions, as TX-MS could confidently locate protein binding interfaces within the repeat regions [39]. Interestingly, the model also proposed that some of the plasma proteins are interacting with other M1-attached human plasma proteins. This observation demonstrates that TX-MS has the capability to determine protein interaction site and the ability to distinguish between direct and indirect protein binding [39]. A highly interesting prospect of additional XL-MS studies is the comparative HP-PPI network protein binding site analysis across species. Such a comparative study could uncover underlying evolutionary conserved interaction patterns [22]. Still, it is clear that additional work is required to further address the quadratic expansion of the computational search space and the unequal fragmentation efficiency of two cross-linked peptides, which typically makes it difficult to unambiguously identify the XL-peptides in complex samples. These developments would allow to more routinely integrate XL-MS workflows in integrative structural biology approaches.

## Cryo-electron microscopy to study bacteria–human interactions

As detailed above, AP-MS and XL-MS can provide detailed information of global HP-PPIs, their composition, dynamic regulation, overall topology and specific protein–protein interaction sites. However, even when combined with structural modeling, XL-MS and AP-MS are unable to provide high-quality, atomic resolution structural information of the individual proteins in a complex or the structure of the complete HP-PPC. For studies where high-resolution structural information is required, XL-MS can be combined with single-particle cryoEM (see “INFO BOX 4”) as described for several large, multicomponent human protein complexes as recently reviewed [41, 51]. To date, the majority of studies on HP-PPCs applying either single-particle cryoEM or cryoET have been performed mostly on virus–host PPCs and PPIs—such as interactions with host receptors [52–55]. Such studies show the potential for equal ones on bacteria–human interspecies PPCs targeting structures at the host–pathogen interface. For bacteria–human HP-PPIs, the examples on electron cryo-microscopy only include cryoET, which, regardless of being a powerful visualization technique, does not provide atomic resolution detail on the PPI interface.

**Table:** 

**Info box 4: Single-particle electron cryo-microscopy and electron cryo-tomography**
In single-particle electron cryo-microscopy (cryoEM), the purified protein or protein complex is preserved in vitreous water on sample grids allowing for their native structural state to be maintained [26]. Imaging of such samples is performed under cryogenic temperatures to protect the specimen from radiation damage. Here, the assumption is that the particles studied obtain random orientations on the sample grid. During imaging, tens of thousands up to million(s) of two-dimensional (2D) projections of individual particles are collected [56, 57]. These 2D projections are aligned and averaged to generate a three-dimensional (3D) reconstruction of the protein or protein complex using dedicated image-processing algorithms [58, 59]. In cases where the 3D structure reaches atomic resolution, the amino acid sequence can be built into the 3D map to generate a 3D model of the protein or protein complex. Single-particle cryoEM is typically applied for macromolecular complexes ranging in size from below 100 kDa (such as hemoglobin [60]) to MDa (such as intact viruses [61]).
In contrast, electron cryo-tomography (cryoET) allows for the three-dimensional visualization of intact cells and cellular structures. The sample(s) studied (intact cells, larger viruses) is preserved in vitreous water on special sample grids allowing for its native structural state to be maintained, much like in single-particle cryoEM. However, during imaging, the sample is rotated within the microscope by tilting the grid along one; sometimes two axes, and a ‘tilt-series’ of two-dimensional (2D) projections are acquired and then used for the calculation of a three-dimensional (3D) reconstruction or tomogram [62]. Due to this imaging technique and the lack of single-particle averaging for a higher signal–noise ratio, the achievable resolution is limited. The resolution is further limited by the thickness of the sample, as the electron beam typically can only penetrate 500 nm into the sample [62]. This means in practice that only prokaryotes can be imaged *in toto,* whereas other cells must be thinned down [63]. Mammalian cells and tissues are often sliced into thinner sections via cryo-sectioning or focused ion beam milling before visualization [25, 64]. The resolution of certain symmetric and repetitive features in the tomogram—such as smaller cellular components or viral surface proteins—can be increased by sub-tomogram averaging [65, 66]. Here, these features are processed as individual protein(s) much like as in single-particle cryoEM, where 2D projections of individual particles are collected, aligned and averaged to generate a 3D reconstruction of the protein or protein complex, with the distinction that in sub-tomogram averaging, the particles are represented by 3D volumes rather than 2D projections.

Much of current state-of-the art work in imagining interactions between bacteria and human via cryoET have been done on *Listeria monocytogenes* [67] and the intracellular *Chlamydia trachomatis* [66, 68, 69]. For example, Nans et al. [68] cultivated human cells directly on sample grids, infected them with *C. trachomatis* elementary bodies (EBs) released naturally from co-cultured infected cells and visualized them by cryoET. In this way, a system was developed to visualize snapshots of *Chlamydial* EBs under physiological conditions during early stage cell entry, including the type III secretion system (T3SS) needles in direct contact with the host plasma membrane [68]. Nans et al. also determined the structure of the T3SS in a host-free environment and in contact with host plasma membrane followed by sub-tomogram averaging, discerning several conformational differences between these two states. Nans et al., thus, revealed that the T3SS acts like a ‘molecular syringe’ during effector protein release into the host-cell cytoplasm [66]. Jasnin et al. used the same approach by cultivating epithelial kidney cells directly on the sample grids, infecting them with *L. monocytogenes* and visualizing the infected cells by cryoET, followed by tomogram interpretation by an automatic segmentation algorithm developed specifically for the tracking of actin filaments [70]. The work by Jasnin et al. proposed a model of actin nucleation and comet tail assembly on the bacterial surface with the bacterial ActA and the human Arp2/3 [71] being the key players, leading to simultaneous polymerization of multiple tangential actin filaments [67, 72].

Although cryoET is an important visualization technique as demonstrated above, it does not provide molecular level detail of the HP-PPIs or HP-PPCs. Sub-tomogram averaging of frequent protein–protein contacts during infection could alleviate this gap in knowledge, much as has been done for the *C. trachomatis* T3SS needle syringe-like movement when injecting effector proteins into the host cytoplasm [66] or in combination with quantitative proteomics [65]. There is still considerable amount of detailed knowledge in infection medicine to be derived from such experiments by exploring new bacteria–human host–pathogen systems.

## Structural modeling approaches

The successful integration of biological mass spectrometry with cryoEM and cryoET will be strongly dependent on novel structural molecular modeling approaches. Structural molecular modeling has changed in recent years by the introduction of low-resolution modeling techniques and fragment-based movers. These techniques use segments of known protein structures to ensure that perturbations to the model simulation adhere to biochemical constraints that determines the three-dimensional structure of the protein [71] (see “INFO BOX 5”). This revolution and the increase of understanding it provides have supported the design of enzymes [73], the creation of new protein topologies [74] and the design of self-assembling proteins [75]. Protein–protein docking and flexible-backbone protein–protein docking can now be carried out routinely as long as experimental structures or high-quality homology models exist [76]. Additionally, molecular modeling in conjunction with XL-MS has allowed us to provide detailed structures of bacteria–human HP-PPCs [39].

**Table:** 

**Info box 5: Molecular modeling**
The first step in modeling the structure of a protein is de novo (ab initio) modeling of the structure from the amino acid sequence without prior knowledge about the spatial arrangement of the amino acids [77]. This approach predicts a protein’s folding based on physical/chemical principles without making use of explicit homolog or template structures in contrast to template-based algorithms. Some successful de novo approaches according to the thirteen Critical Assessment of Techniques for Protein Structure Prediction (CASP13) include MULTICOM [78], SWISS-MODEL [79], QUARK [80], and Rosetta [81].
Predicting the structure of a protein can also be addressed by comparative modeling approaches when there is a suitable template or homologous structure that can be used to guide the process. Comparative modeling approaches mainly align the sequence of two (or more) proteins and use the template structure(s) for the similar parts and try to model the gaps by de novo modeling or other fragment-based approaches. In this category, some of the popular softwares are RosettaCM [82], Modeler [83], HHpred [84], and I-TASSER [85].
In addition to the aforementioned methods, models or low-resolution experimental data from NMR or X-ray crystallography can be improved and refined by several computational techniques such as loop-modeling approaches like next-generation KIC (NGK) [86], and DaReUS-Loop [87, 88] or experimental data-based protocols of Rosetta such as RosettaES [89], CS-Rosetta [90], and RosettaNMR [91].

## Future directions—towards an integrative approach in bacteria–human interactions

With current state-of-the-art instrumentation, the constantly improving data-processing algorithms and bioinformatics tools—particularly in MS and electron microscopy—there is a vast possibility of combining quantitative and structural mass spectrometry with advanced structural biology. Such methods could encompass AP-MS, XL-MS, hydrogen–deuterium exchange mass spectrometry (HDX-MS), native mass spectrometry, single-particle cryoEM, X-ray crystallography, NMR, small angle X-ray and neutron scattering methods, together with cellular visualization methods (foremost cryoET but also, e.g., correlative light and electron microscopy). As mentioned earlier, such integrative studies would advance our understanding in pathogenesis, yet a lot remains to be done with respect to applying such approaches in medical microbiology. Current studies have combined electron cryo-microscopy, XL-MS and structural modeling to a large extent to understand intra-species, i.e., human–human protein complexes [40, 92–97].

In this review, we have showcased different techniques involved in understanding different tiers in HP-PPI. Integrative approaches involving AP-MS, XL-MS, cryoEM and structural modelling could provide combined knowledge and valuable insights into the process of infection. AP-MS identifies interacting protein pairs; however, it fails to identify the interacting peptides and domains between proteins. AP-MS coupled with XL-MS would not only identify interacting proteins, but also stabilize transient interactions and identify amino acid pairs between the interacting proteins. Complementing AP-MS and XL-MS with single-particle cryoEM would provide additional structural information on the complete HP-PPC. Both XL-MS and single-particle cryoEM require small sample amounts and both can be applied to heterogeneous samples. For larger protein complexes, where the local resolution in cryoEM maps can vary considerably with usually highly defined core regions and more poorly resolved densities towards the edges, XL-MS can help in resolving the structure of these edges by providing the needed distance constraints between proteins or their domains [51]. However, whereas it is difficult to determine via XL-MS whether a cross-link between peptides arises from a more or less populated protein (complex) conformation, this information can be determined via single-particle cryoEM by classification particles and derived volumes during data processing [51, 98]. Other limitations of XL-MS relate to possible unequal fragmentation efficiency of two cross-linked peptides and identification of sparse networks of cross-linked distance constraints. Incorporating structural modeling approaches such as de novo modeling, comparative modeling, and protein–protein docking will play an important role in overcoming these limitations. TX-MS [39] as mentioned above is a successful example of such combination, as it overcomes the aforementioned limitations and enables the structural modeling of large macromolecular assemblies with dense networks of distance constraints. The usefulness of XL-MS to structural, proteome-wide studies will further be pushed by recent developments in cross-linkers [43] and structural modeling and docking approaches [14, 39, 43]. It can further be anticipated that integrative structural approaches in medical microbiology will be beneficial for the design of novel therapeutic approaches. For example, the emerging field of structure-based design of vaccines—also referred to as structural vaccinology—has recently started to deliver new vaccine antigens [99]. Structural vaccinology aims at optimizing protective B-cell epitopes using a combination of X-ray, electron microscopy, mass spectrometry and computational approaches [100]. Pioneering studies have shown that HP-PPI between human proteins and bacterial proteins influences protective antibody responses [101, 102], which is of importance for rational design of cross-protective antigens [103]. Furthermore, efficient physical and structural epitope mapping abilities using for example XL-MS, HDX-MS, X-ray crystallography and more recently cryoEM for large antigens provides essential information for antigen engineering to guide vaccine design and optimization [100]. Such information in combination with human B-cell repertoire sequence analysis represents a promising way forward for investigations regarding the structural basis for epitope immunodominance and the use of such information in vaccine design [99]. Further technological developments in this area will enable us to enhance our understanding of the structural basis for HP-PPI and immunogenicity to further improve vaccine efficiency and potentially other targeted treatment strategies [104].

## Concluding remarks

In this review, our focus is on demonstrating how AP-MS, XL-MS, cryoET and molecular modeling have been successfully used to address the organization and dynamics of HP-PPI networks and the structure of HP-PPCs. Incorporating integrative structural biology more broadly in infection medicine research would increase our understanding of how the diverse HP-PPIs facilitate bacterial adhesion, dissemination and survival within the host. These efforts could also have a future impact on drug development programs to interfere with the interaction and assembly of these HP-PPCs as well as for vaccine development strategies to combat these infections.
